# Enhanced tolerance to Phytophthora root and stem rot by over-expression of the plant antimicrobial peptide *CaAMP1* gene in soybean

**DOI:** 10.1186/s12863-020-00872-0

**Published:** 2020-07-06

**Authors:** Lu Niu, Xiaofang Zhong, Yuanyu Zhang, Jing Yang, Guojie Xing, Haiyun Li, Dongbo Liu, Rui Ma, Yingshan Dong, Xiangdong Yang

**Affiliations:** 1 Jilin Provincial Key Laboratory of Agricultural Biotechnology, Jilin Academy of Agricultural Sciences, Changchun, 130033 China; 2 Jilin Normal University, Siping, 136000 China

**Keywords:** *CaAMP1*, Transgenic soybean, PRR tolerance, Antimicrobial peptide

## Abstract

**Background:**

Antimicrobial peptides play important roles in both plant and animal defense systems. Moreover, over-expression of *CaAMP1* (*Capsicum annuum antimicrobial protein 1*), an antimicrobial protein gene isolated from *C. annuum* leaves infected with *Xanthomonas campestris* pv. *vesicatoria*, confers broad-spectrum resistance to hemibiotrophic bacterial and necrotrophic fungal pathogens in *Arabidopsis*. Phytophthora root and stem rot (PRR), caused by the fungus *Phytophthora sojae*, is one of the most devastating diseases affecting soybean (*Glycine max*) production worldwide.

**Results:**

In this study, *CaAMP1* was transformed into soybean by *Agrobacterium*-mediated genetic transformation. Integration of the foreign gene in the genome of transgenic soybean plants and its expression at the translation level were verified by Southern and western blot analyses, respectively. *CaAMP1* over-expression (*CaAMP1-*OX) lines inoculated with *P. sojae* race 1 exhibited enhanced and stable PRR tolerance through T_2_–T_4_ generations compared with the wild-type Williams 82 plants. Gene expression analyses in the transgenic plants revealed that the expression of salicylic acid-dependent, jasmonic acid-dependent, and plant disease resistance genes (*R*-genes) were significantly up-regulated after *P. sojae* inoculation.

**Conclusions:**

These results indicate that *CaAMP1* over-expression can significantly enhance PRR tolerance in soybean by eliciting resistance responses mediated by multiple defense signaling pathways. This provides an alternative approach for developing soybean varieties with improved tolerance against soil-borne pathogenic PRR.

## Background

More than 8000 antimicrobial peptides (AMPs), representing key components of the innate immune system in both plants and animals, have been isolated from amphibians, mammals, plants, bacteria, and insects [1]. Plant AMPs are expressed in roots, stems, leaves, flowers and seeds, and inactivate prokaryotic cells by targeting several essential metabolic processes at extracellular and intracellular sites and the plasma membrane [2]. AMPs are known to play important roles in constitutive or induced resistance to various pathogens, by degrading fungal cell walls, inducing membrane channel and pore formation, inhibiting DNA synthesis and cell cycle, and damaging cellular ribosomes [3–7]. Plant AMPs have been shown to enhance tolerance to many fungal diseases in several species, including pepper [8], rice [7, 9, 10], potato [11], tobacco [3], and creeping bentgrass and citrus [12, 13]. In addition, a defensin from chickpea, which is a type of AMP, confers tolerance against water deficit stress in *Arabidopsis thaliana* [14].

*CaAMP1*, an AMP gene isolated from pepper (*C. annuum*) leaves infected with *Xanthomonas campestris* pv. *vesicatoria*, has been implicated in broad-spectrum resistance to the hemibiotrophic bacterial pathogen *Pseudomonas syringae* pv. *tomato*, biotrophic oomycete *Hyaloperonospora parasitica*, and fungal necrotrophic pathogens *Fusarium oxysporum* f. sp. *matthiolae* and *Alternaria brassicicola* [8]. Over-expression of *CaAMP1* enhances tolerance to hemibiotrophic bacterial and necrotrophic fungal pathogens in *Arabidopsis*, when compared with the wild-type plants, and induces two salicylic acid (SA) pathway-dependent genes, i.e., *PR1* (*PATHOGENESIS-RELATED*) and *PR5* expression [8]. Defensin genes isolated from rice (*OsDEF7* and *OsDEF8*) have been shown to inhibit the phytopathogens *X. campestris* pv. *glycines*, *X. oryzae* pv. *oryzicola*, and *Erwinia carotovora* subsp. *atroseptica*, and weaken the activity of the phytopathogenic fungi *Helminthosporium oryzae* and *F. oxysporum* f. sp. *cubense* [7, 9]. Protein extracts with MSI-99, an AMP expressed in chloroplasts of tobacco, could significantly suppress two rice blast isolates, both in vitro and in vivo [10]. Furthermore, leaf extracts from transplastomic tobacco are shown to inhibit the growth of pregerminated spores of three fungal species, *Aspergillus flavus*, *F. moniliforme*, and *Verticillium dahliae* [3], whereas expression of the AMP *alfAFP* in transgenic greenhouse-grown potato confers tolerance against *V. dahliae*, an agronomically important fungal pathogen [11]. In addition, AMPs have also been demonstrated to enhance tolerance to fungal diseases in bentgrass and citrus [12, 13]. Collectively, these findings evidence that AMPs play key roles in plant defense against fungal pathogens, and that over-expression of these peptides can enhance tolerance against many fungal diseases.

Soybean (*Glycine max* L. Merr.) is an economically important crop worldwide, acting as a rich source of vegetable oil and protein for both humans and livestock. Phytophthora stem and root rot (PRR), caused by the soil-borne hemibiotrophic oomycete *Phytophthora sojae*, is one of the most devastating fungal diseases in soybean, resulting in an annual yield reduction and economic loss of approximately 10–50% and 1–2 billion dollars, respectively [15–18]. PRR caused by *P. sojae* has become a major threat to soybean production in China since it was first reported in 1989 [15, 19]. Current measures for controlling PRR in the fields include drainage improvement, crop rotation, and fungicide application. Host-mediated resistance provided by “resistant to *P. sojae*” (*Rps*) genes has also been employed to improve PRR tolerance in soybean [16, 20–25]. However, *Rps*-mediated resistance to PRR in soybean may be lost over long periods owing to the high variability of *P. sojae*, with at least 55 races identified to date [26]. Other alternative control methods include the development of transgenic soybean with enhanced PRR tolerance achieved by the increased levels of pathogenesis-related (PR) proteins, such as Gly m 4 l and ethylene response factor [27], or harpin protein-encoding genes [28, 29].

As mentioned previously, AMP over-expression confers broad-spectrum resistance against bacterial and fungal pathogenic infections in plants, suggesting that AMPs may enhance soybean resistance to PRR. Therefore, we synthesized *CaAMP1* and introduced it into soybean via *Agrobacterium*-mediated transformation, and then evaluated the tolerance of *CaAMP1-*OX soybean to PRR. We found that over-expression of *CaAMP1* enhanced soybean tolerance to PRR, and induced the expression of genes involved in SA- and JA-dependent pathways and *R*-gene signaling.

## Results

### Generation and screening of transgenic plants

**Fig. 1:**
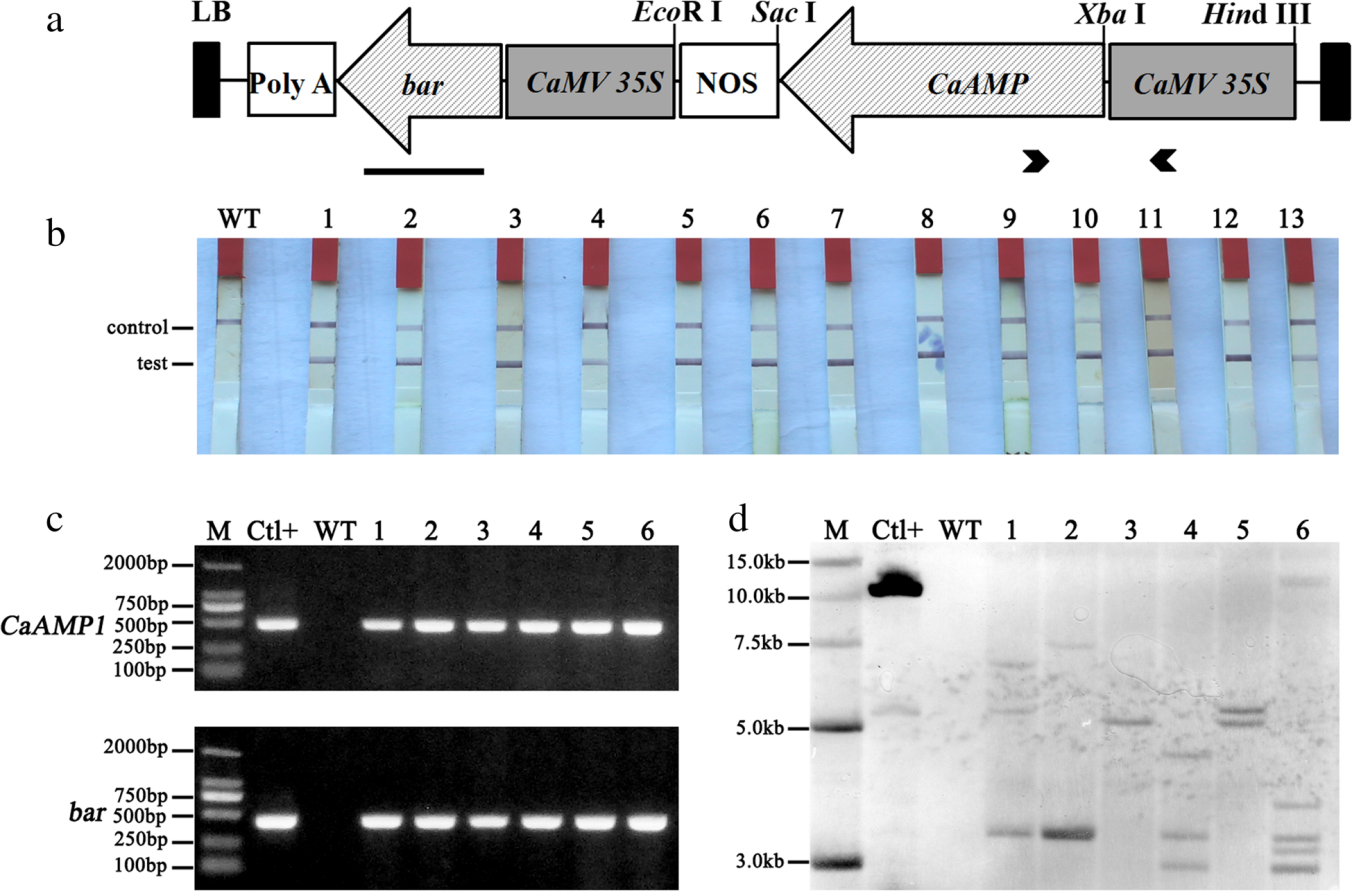
Schematic representation of the plant expression vector and screening of transgenic soybean. (A) Schematic representation of the recombinant plasmid pCambia3300-CaMV 35S-CaAMP1. RB and LB, the right and left borders of the T-DNA, respectively. CaAMP1 and bar inserts were both driven by the CaMV 35S promoter. Bold arrows indicated the primer binding sites of CaAMP1-F1/R1. The solid bar represented the PCR product using Bar-F/R. (B) LibertyLink strip analysis of the T0 transgenic plants. WT, wild-type Williams 82; 1–13, transgenic soybean lines. (C) PCR analysis of T3 transgenic lines using CaAMP1-F1/R1 and Bar-F/R primers. M, DL2000 marker; Ctl+, positive plasmid; WT, wild-type Williams 82; numbers 1–6, transgenic soybean lines 8096, 8101, 8111, 8130, 8197, and 8253, respectively. (D) Southern blot analysis of the T3 transgenic lines using CaAMP1 as a probe. M, 15-kb DNA marker; Ctl+, positive plasmid; WT, wild-type Williams 82; 1–6, transgenic lines 8096, 8101, 8111, 8130, 8197, and 8253, respectively

The nucleotide sequence of the *CaAMP1* (GenBank ID: AAT35532.1) was synthesized and subcloned into a pCambia3300 vector (Fig. 1a). Constitutive expression of *CaAMP1* was induced in the soybean plants under the control of *CaMV 35S* promoter. Constitutive expression was desirable because *P. sojae* can infect soybean at various developmental stages in much of the growing season. Transgenic soybean was generated via *Agrobacterium*-mediated transformation, with the Williams 82 cultivar as the recipient.

A total of 185 phosphinothricin *N*-acetyltransferase (PAT)-tolerant plants were generated and screened using the LibertyLink strip test, and the positive ones (indicated by two red lines in Fig. 1b) were grown in a greenhouse to produce seeds. Six T_3_ transgenic soybean lines were selected for PCR and Southern blot analyses. The PCR confirmed that T_3_ transgenic soybean lines contained *CaAMP1* and *bar* genes (Fig. 1c). Southern blot analysis using *CaAMP1* as the probe further confirmed that *CaAMP1* was integrated into the genome of transgenic soybean, with approximately 1 to 5 copies of insertions (Fig. 1d). The size of all detected bands was greater than the expected fragment size of 1.98 kb, which covered the sequence between the right border and the unique *Eco*R I site. In contrast, no signal was detected in the wild-type Williams 82 plants. These results indicated stable integration of *CaAMP1* in transgenic soybean.

**Fig. 2:**
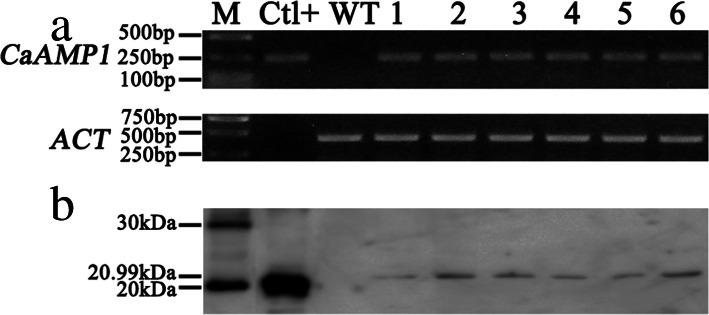
Analysis of CaAMP1 expression in transgenic soybean lines. (A) RT-PCR analysis of the transgenic lines. (B) Western blot analysis of the transgenic lines. M, DL2000 DNA marker (A) & protein ladder (B); Ctl+, positive plasmid; WT, wild-type Williams 82; numbers 1–6, T3 transgenic lines 8096, 8101, 8111, 8130, 8197, and 8253, respectively

RT-PCR and western blot analyses were further performed to detect *CaAMP1* expression in the six T_3_ transgenic soybean lines. A 275-bp fragment was detected in all six transgenic lines by RT-PCR, which was absent from the wild-type Williams 82 (Fig. 2a). We also detected 20.99 kDa bands in the six transgenic lines, which was absent from the wild-type plants, confirming the expression of *CaAMP1* at both transcriptional and translational levels in the transgenic soybean (Fig. 2b). These results indicated that *CaAMP1* was successfully transformed into the soybean, and accurately transcribed and translated in the six transgenic lines.

### Stable and enhanced tolerance of transgenic soybean to PRR

**Fig. 3:**
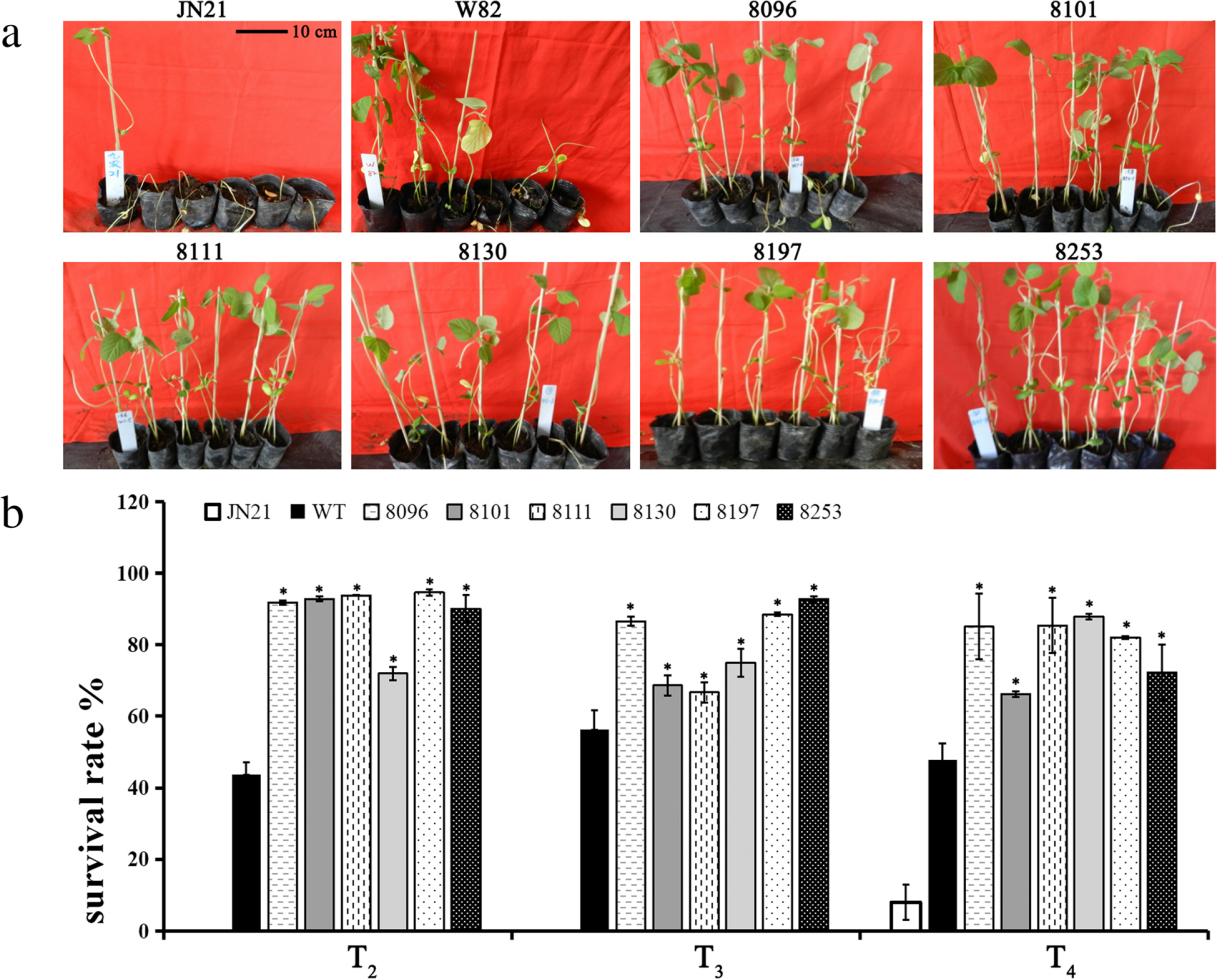
Tolerance of T2–T4 transgenic lines to Phytophthora root and stem rot under greenhouse conditions. (A) Tolerance responsed 10 days after inoculation with P. sojae race 1. JN21, susceptible cultivar Jiunong 21; W82, wild-type Williams 82; 8096 to 8253, T3 transgenic lines. (B) Survival rate of three generations of transgenic lines inoculated with P. sojae race 1. All experiments were replicated thrice with 20 inoculated plants per replicate. The average survival rate of each transgenic line was calculated 7 days after inoculation. Error bars indicated standard errors and asterisks denoted significant differences between the transgenic line and the corresponding WT plants at *P* < 0.01

The PRR tolerance of T_3_ transgenic lines and wild-type soybean was evaluated after inoculation of greenhouse-grown plants with *P. sojae* race 1. As shown in Fig. 3, the tolerance of transgenic lines to PRR was markedly enhanced, when compared with that of the wild-type control and Jiunong 21 (JN21) plants which was susceptible to *P. sojae* (Fig. 3). Typical symptoms of PRR were visible in wild-type Williams 82 and JN21 5 to 10 days after inoculation with *P. sojae* race 1 mycelia, with some plants succumbing to the progressing pathogenic infection (Fig. 3a), which was more prominent in JN21. In contrast, most transgenic lines were only slightly affected, as evident from the development of chlorotic leaves (Fig. 3a). Survival rates of transgenic lines (66.17–94.68%) over three generations were higher than those of wild-type Williams 82 (43.67–56.17%) and JN21 (0–8.08%), which was consistent with the results of PRR tolerance (Fig. 3b). Of the six transgenic lines, four (8096, 8101, 8197, and 8253) exhibited a stable enhancement in the tolerance to *P. sojae* race 1, when compared with the JN21 and wild-type controls (Fig. 3b). In conjunction, these results suggest that *CaAMP1-*OX transgenic soybean were more tolerant to PRR.

### Up-regulation of disease-responsive genes in *CaAMP1-*OX transgenic soybean

**Fig. 4:**
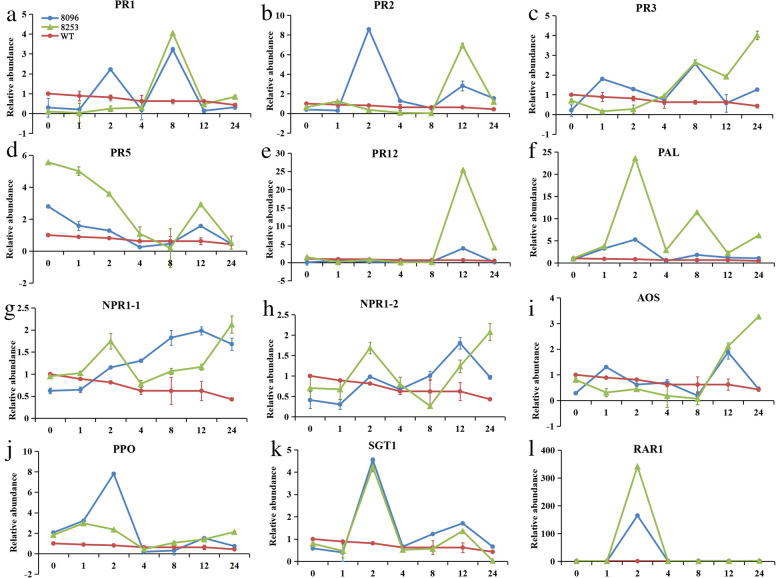
Expression levels of selected defense-responsive genes in transgenic soybean in response to P. sojae inoculation. Total RNA was extracted from fully grown leaves of T3 transgenic plants 0, 1, 2, 4, 8, 12, and 24 h after inoculation with P. sojae race 1 mycelia. Expression level of each gene was quantified relative to that of the internal control (GmACT) using the 2-ΔΔCt method. Data were represented as the mean of three biological replicates, with error bars indicating standard errors. (A) to (H) indicated the expression levels of SA-dependent pathway genes GmPR1, GmPR2, GmPR3, GmPR5, GmPR12, and GmPAL, respectively; (I) and (J) indicate the expression levels of SA-dependent pathway genes GmAOS and GmPPO, respectively; (K) and (L) indicated plant disease resistance genes GmSGT1 and GmRAR1, respectively. WT, wild-type Williams 82; 8096 and 8253, T3 transgenic lines

*CaAMP1* has been shown to induce *PRs* expression in transgenic *Arabidopsis* [8]. In the present study, the transcription of 15 defense-related genes involved in SA- and JA-dependent pathways, and *R*-genes was assessed in two transgenic lines (8096 and 8253) after inoculation with *P. sojae* race 1. The expression levels of SA-dependent pathway genes *GmPR1*, *GmPR2*, *GmPR3*, *GmPR5*, *GmPR12*, *GmPAL*, and *GmNPRs*, were significantly higher in the two transgenic lines than in the wild-type soybean (Fig. 4a–h). Two JA-dependent pathway genes, *GmAOS* and *GmPPO*, also exhibited increased expression in these lines (Fig. 4i and j). Moreover, the expression of both *R*-genes, i.e., *GmSGT1* and *GmRAR1* (Fig. 4k and l), involved in plant resistance to disease, was significantly up-regulated in comparison to the wild-type control. These results indicated that over-expression of the *CaAMP1* gene in soybean could elicit multiple resistance responses mediated by different signaling pathways, enhancing plant tolerance against *P. sojae* infection.

### No obvious differences in agronomic traits between *CaAMP1-*OX and wild-type soybean

To evaluate the effect of over-expression of *CaAMP1* in soybean, agronomic traits, including maturity period, leaf shape, flower color, hilum color, plant height, branch number, node number, podding height, and 100-seed weight, of field-grown T_3_ transgenic lines were analyzed. No differences could be detected in these traits between the transgenic lines and wild-type Williams 82 (Table 1). These results indicated that *CaAMP1* over-expression conferred transgenic soybean with enhanced and stable tolerance to PRR without any negative effects on its agronomic traits.

**Table 1:** Agronomic traits of *CaAMP1-*OX soybean in the fields

Phenotype	Williams 82	8096	8101	8111	8130	8197	8253
Maturity period (days)	131	130	131	130	131	131	130
Leaf shape	Round	Round	Round	Round	Round	Round	Round
Flower color	White	White	White	White	White	White	White
Hilum color	Black	Black	Black	Black	Black	Black	Black
Plant height (cm)	97.76 ± 4.40	97.03 ± 3.74	97.00 ± 4.10	97.13 ± 4.47	96.84 ± 2.87	96.89 ± 4.03	97.19 ± 2.64
Node number	20.20 ± 0.98	20.15 ± 1.01	19.85 ± 1.31	20.25 ± 1.04	19.95 ± 1.20	20.1 ± 0.94	20.25 ± 0.89
Branch number	4.15 ± 0.36	4.20 ± 0.40	4.25 ± 0.433	4.25 ± 0.433	4.10 ± 0.30	4.30 ± 0.46	4.20 ± 0.40
Podding height (cm)	5.41 ± 0.40	5.49 ± 0.41	5.37 ± 0.36	5.35 ± 0.36	5.41 ± 0.400	5.41 ± 0.32	5.44 ± 0.47
100-seed weight (g)	18.91 ± 0.93	18.47 ± 0.81	18.27 ± 0.57	18.01 ± 0.41	18.13 ± 0.39	18.24 ± 0.61	18.19 ± 0.59

Data on the agronomic traits of wild-type Williams 82 and the six transgenic lines (8096, 8101, 8111, 8130, 8197, 8253) were collected from the experimental station at Gongzhuling, Jilin Province, China. Twenty plants of each line were randomly selected for the measurements. Differences were considered significant at *P* < 0.05.

## Discussion

PRR is one of the most devastating diseases in soybean, resulting in annual losses of 1–2 billion dollars worldwide [15, 16]. Previous studies have confirmed that exogenous resistant genes can enhance PRR tolerance in soybean [28, 29]. In the present, an AMP gene, *CaAMP1*, which has previously been demonstrated to confer broad-spectrum resistance against hemibiotrophic bacterial and necrotrophic fungal pathogens in transgenic *Arabidopsis* [8], is transformed into the soybean genome. We obtain six transgenic lines with enhanced tolerance against *P. sojae* race 1 over three generations, compared with the wild-type plants. Furthermore, we observe an up-regulation of several defense-related genes in these transgenic lines. These observations indicate that *CaAMP1* plays a functional role in stimulating defense-related genes involved in SA-dependent, JA-dependent, and *R*-defense signaling in response to pathogenic infection.

SA- and JA-dependent signaling pathways are essential for defense against pathogens. SA is crucial to immune responses against biotrophic and hemibiotrophic pathogens [30, 31], and also involves in cross talks between ethylene and methyl jasmonate signaling pathways [32, 33]. SA-dependent defense pathways can be induced when pathogen infects the plant and finally activate on pathogenic infection in plants, subsequently activating the *PR*s [34]. Over-expression of CaAMP1 protein in *Arabidopsis* triggers rapid expression of *AtPR1* and *AtPR5* after infection with the virulent strain *Pst* DC3000, enhancing plant resistance to this disease [8]. In addition to *PR* genes, three other SA-dependent pathways genes, i.e., *PAL*, *NPR1–1*, and *NPR1–2* are also up-regulated in the *CaAMP1* transgenic lines in the present study. *PAL* is required in SA bio-synthesis pathways [35–37], and *NPRs* are indispensable for cross talks between SA and JA/ethylene responses [38]. Over-expression of these two genes can enhance disease tolerance in soybean and *Arabidopsis* [39, 40]. Furthermore, the activation of SA-dependent *PR* genes is observed to be more rapid and intense in *NPR1*-OX transgenic plants than in their wild-type counterparts [40]. These genes can also be up-regulated in transgenic soybean by exogenous oxalate oxidase, *hrpZm*, and *hrf2*, after infection with *P. sojae* race 1 [28, 29, 37].

JA-regulated defense is an important component of plant resistance to necrotrophic fungi [32, 41, 42]. *AOS* and *PPO*, involved in JA-dependent signaling pathway, are induced in *CaAMP1*-OX soybean lines. This is not consistent with the observations made in *Arabidopsis* [8], which may be attributed to the different genes selected in the present study. *SGT1* and *RAR1* are important genes in plant resistance signaling pathways [43–45], which are also up-regulated in *CaAMP1*-OX transgenic soybean.

Collectively, these results suggest that CaAMP1, as an AMP, can enhance PRR tolerance in transgenic soybean by triggering the production of SA- and JA-dependent defense signaling molecules and *R*-genes. These results confirm that rapid induction of SA and JA signaling pathways is associated with early recognition of the pathogen and resistance in soybean [46]. Furthermore, we do not observe differences in the agronomic traits between *CaAMP1-*OX lines and their wild-type counterparts.

## Conclusions

The results of the present study indicated that over-expression of *CaAMP1* in soybean can significantly enhance PRR tolerance by inducing the expression of resistant genes involved in multiple defense signaling pathways. This may provide an alternative approach for developing soybean varieties with improved tolerance against soil-borne pathogenic PRR.

## Methods

### *CaAMP1* synthesis and vector construction

The nucleotide sequence of *C. annuum CaAMP1* (GenBank ID: AAT35532.1) was synthesized with added *Xba* I and *Sac* I recognition sites at the 5′ and 3′ ends, respectively (Sangon Biotech, Shanghai, China). The modified *CaAMP1* gene was inserted into a pCambia3300 vector containing a modified *CaMV 35S* promoter [47] (GenBank: GI3319906) to facilitate its constitutive expression in soybean. The gene sequence was amplified using the *CaAMP1*-F/R primer pair, with a final primer concentration of 0.4 μM, under the following conditions: 94 °C for 5 min; followed by 35 cycles of 94 °C for 30 s, 59 °C for 30 s, and 72 °C for 30 s; and final extension at 72 °C for 7 min. All primers used in this study were listed in Table S1. The purified fragment was then subcloned into a pCambia3300 plasmid containing a phosphinothricin acetyl transferase (*bar*) resistance gene, encoding PAT, as a plant selection marker driven by a modified *CaMV 35S* promoter [47] (GenBank: GI3319906). The constructed pCambia3300-*CaMV 35S*-*CaAMP1* plasmid was subsequently transformed into competent *A. tumefaciens* strain EHA101 cells, by the freeze-thaw method [48, 49].

### Regeneration and screening of transgenic plants

*Agrobacterium*-mediated transformation was used for regenerating transgenic soybean, with the soybean cultivar Williams 82 as the recipient, which was provided by Prof. Fudi Xie of Shenyang Agricultural University, China (ID: WDD00587, Chinese Crop Germplasm Information System, http://www.cgris.net), following the method described in Yang et al. (2018) and Zhang et al. (2014) [50, 51]. The regenerated PAT-tolerant plants were screened using LibertyLink® strip test (cat #AS 013 LS; EnviroLogix Inc., Portland, ME, USA) and PCR. Herbicide-tolerant T_1_–T_3_ transgenic lines were identified by spraying the leaves with 500 mg∙L^− 1^ glufosinate (EnviroLogix Inc., Portland, Maine, USA) on complete expansion of the first trifoliate leaves, and then analyzed by PCR using the *CaAMP1*-F1/R1 and *Bar*-F/R primer pairs (Table S1) until homozygous transgenic plants were obtained. DNA was extracted from the leaves of transgenic and wild-type soybean, using a simple homogenization and ethanol precipitation method, for PCR analysis [52]. PCR was performed with a final primer concentration of 0.2 μM, with the following conditions: 94 °C for 5 min; followed by 35 cycles of 94 °C for 30 s, 58 °C for 30 s, and 72 °C for 30 s; and final extension at 72 °C for 7 min.

To confirm the integration of T-DNA in transgenic soybean, T_2_ transgenic plants were selected for genomic DNA extraction, using a modified high salt cetyl-trimethyl ammonium bromide method [53]. DIG High Prime DNA Labeling and Detection Starter Kit I (No. 11745832910; Roche Applied Science, Indianapolis, IN, USA) was used for Southern blot analysis, according to the manufacturer’s instructions. Approximately 30 μg of the genomic DNA from transgenic soybean and control plants was digested completely with *Eco*R I (New England Biolabs Inc., Beverly, Massachusetts). The digested DNA was then transferred onto positively charged nylon membranes (GE Amersham, RPN303B, USA). Hybridization was carried out at 42 °C for 12–16 h, using *CaAMP1* labeled with digoxigenin-(DIG)11-dUTP as a probe. The washing conditions and signal detection were as described in Yang et al. (2018) [50].

### Expression analysis in transgenic soybean

Total RNA and proteins were extracted for expression analysis. Total RNA was extracted from 2-week-old leaves of T_3_ transgenic plants (8096, 8101, 8111, 8130, 8197, 8253) using a EasyPure PlantRNA Kit (TransGen Biotech, Beijing, China), and DNase I was used to eliminate the contaminant genomic DNA. cDNA was then synthesized using the ThermoScript RT-PCR system (Invitrogen, USA), and RT-PCR was performed using CaAMP-RF/RR primers (Table S1). *GmACT* (GeneBank ID: NM 001289231), amplified using the primers 5′-CACCGGAGTTTTCACCGATA-3′ and 5′-AGGAATGATGTTAA-3′, was used as the control.

Crude proteins were extracted from ~ 100 mg fresh leaves of the control and T_3_ transgenic soybean lines (8096, 8101, 8111, 8130, 8197, 8253), separated on a 12% (w/v) SDS-PAGE gel, and then transferred electrophoretically onto a PVDF membrane (Amersham™ Hybond™, GE Healthcare, USA) [54]. After blocking with 3% dried skimmed milk diluted in PBST (1× PBS, 0.1% Tween-20), the membrane was blotted with a rabbit polyclonal antibody (1:500 dilution) raised against recombinant CaAMP1 protein (GenScript Co., Ltd. Nanjing, China) and horseradish peroxidase (HRP)-labeled goat-anti-rabbit IgG (1:5000 dilution; Abcam, UK) at 25 °C for 4 h. The bands observed following western blotting were visualized using the BiodlightTM Western Chemiluminescent HRP substrate (Bioworld Technology, Inc., St. Louis, MN, USA) after extensive washing.

### Evaluation of PRR tolerance under greenhouse conditions

To evaluate the tolerance of transgenic soybean against *P. sojae* race 1, the T_2_ − T_4_ generations of transgenic lines 8096, 8101, 8111, 8130, 8197, and 8253 were infected with *P. sojae* race 1, following the method described by Schmitthenner et al. (1994) [55]. Isolation and cultivation of the inoculum were performed as described by Akamatsu et al. (2010) and Du et al. (2018) [28, 56]. Transgenic soybean, wild-type Williams 82, and the PRR-susceptible cultivar Jiunong 21 (ID:ZDD22796), which were provided by the Soybean Research Institute of Jilin Academy of Agricultural Sciences, were grown in a greenhouse, and the hypocotyls of 15-day-old seedlings were inoculated with macerated mycelia of *P. sojae* race 1. The plants were then maintained in a humid environment for 15–24 h, before being transferred to the greenhouse for symptom development, at 25 °C under an 18-h light/6-h dark photoperiod [28]. After 5 to 10 days of inoculation, plant infection data were collected and survival rates were calculated [57]. All experiments were performed with three replicates of 20 inoculated plants each replicate.

Differences in the survival rates of the control and transgenic lines were quantitatively assessed by *t-*test at a significance level of *P* = 0.05 or 0.01, using Microsoft Analysis Tool.

### Quantitative RT-PCR analysis of disease-responsive genes

Leaves were collected from T_3_ transgenic lines (8096 and 8253) and wild-type Williams 82 plants, 0, 1, 2, 4, 8, 12, and 24 h after inoculation with *P. sojae* race 1 mycelia, for quantitative PCR. Total RNA extraction and cDNA synthesis were performed as described in previous sections. The relative expression levels of 12 genes involved in different stress response pathways, including *GmPR1* (AF136636), *GmPR2* (M37753), *GmPR3* (AF202731), *GmPR5* (BU765509), *GmPR12* (BU964598), *GmPAL* (X52953), *GmPPO* (EF158428), *GmAOS* (DQ288260), *GmSGT1* (NM_001249656), *GmNPR1–1* (FJ418594), *GmNPR1–2* (FJ418596), and *GmRAR1* (FJ222386), were analyzed by qRT-PCR, with *GmACT* (U60500) as the internal control. Amplification was performed in a final reaction volume of 20 μL, with ~ 80 ng cDNA and 0.4 μL each of forward and reverse primers (Table S1), using a SYBR Green-based One-Step qRT-PCR kit (TransGen Biotech, China). The conditions for the qRT reaction were as follows: 50 °C for 2 min; 95 °C for 10 min; and 45 cycles of 95 °C for 2 min, 62 °C for 30 s, and 72 °C for 30 s. The relative expression level of each gene was determined using the 2^-ΔΔCt^ method [58]. To improve the accuracy of the data, three biological and three technical replicates were performed for each experiment.

### Agronomic traits of transgenic lines

Nine agronomic traits of T_3_ transgenic lines and wild-type soybean were assessed, including maturity period, leaf shape, plant height, flower color, hilum color, branch number, node number, podding height, and 100-seed weight, and *t-*test was used for quantitative analysis.

## Supplementary information

**Additional file 1.** Supplementary Table 1

## Data Availability

All datasets used and/or analyzed in this study are available from the corresponding authors on reasonable request.

## Abbreviations

CaAMP1: *Capsicum annuum* antimicrobial protein 1
PRR: Phytophthora root and stem rot
SA: Salicylic acid
JA: Jasmonic acid
AMPs: Antimicrobial peptides
*PR*: *Pathogenesis-related*
*OsDEF7*: *Oryza sativa defensin 7*
*OsDEF8*: *Oryza sativa defensin 8*
OX: Over-expression
*CaMV 35S*: Cauliflower mosaic virus 35S promoter
PCR: Polymerase chain reaction
PAT: Phosphinothricin N-acetyltransferase
EDTA: Ethylene diamine tetraacetic acid
SDS: Sodium dodecyl sulfonate
PVDF: Polyvinylidene fluoride
PBST: Phosphate buffered solution with Tween-20
HRP: Horseradish peroxidase
*PAL1*: Phenylalanine ammonia lyase 1
*PPO*: Polyphenol oxidase
*AOS*: Allene oxide synthase
*SGT1*: Suppressor-of-G2-allele-of-skp 1
*NPR1*: Nonexpressor of pathogenesis-related gene 1
*RAR1*: Required for Mla12-mediated resistance 1
*ACT*: Actin
RT: Reverse transcript
