# Supplementary material for: Enhanced tolerance to Phytophthora root and stem rot by over-expression of the plant antimicrobial peptide CaAMP1 gene in soybean
Source: BMC Genet. 2020 Jul 6;21:68. doi: 10.1186/s12863-020-00872-0 (PMC7336493; doi:10.1186/s12863-020-00872-0)
Supplement: Supplementary file 1 — Additional file 1. Supplementary Table 1 [file 12863_2020_872_MOESM1_ESM.docx]

Supplementary Table 1

| Genes | GenBank No. | Forward/reverse primers |
| --- | --- | --- |
| *CaAMP1-*F/R | [AAT35532.1](https://www.ncbi.nlm.nih.gov/protein/AAT35532.1?report=genbank&log$=prottop&blast_rank=1&RID=C5PFZSJF01R) | 5′-GCTCTAGAATGAATGCTAATGGATTTAGCGGTG-3′ |
|  |  | 5′-GAGAGCTCTTAGACCTGATCAATGGGTTCTGTC-3′ |
| *bar* | GI3319906 | 5′-GTGGATTGATGTGATATCTCCACT-3′ |
|  |  | 5′-GAGGAGTAGTTCCTAAGTCTGTGATC-3′ |
| *CaAMP1-*F1/R1 | [AAT35532.1](https://www.ncbi.nlm.nih.gov/protein/AAT35532.1?report=genbank&log$=prottop&blast_rank=1&RID=C5PFZSJF01R) | 5′-GTGGATTGATGTGATATCTCCACTG-3′ |
|  |  | 5′-GAGGAGTAGTTCCTAAGTCTGTGATG-3′ |
| *CaAMP1-*RF/RR | [AAT35532.1](https://www.ncbi.nlm.nih.gov/protein/AAT35532.1?report=genbank&log$=prottop&blast_rank=1&RID=C5PFZSJF01R) | 5′-ATGCCAGTTCCCGTGCTTGAAG-3′ |
|  |  | 5′-CTAGGGGGATCTACCATGAGCCCA-3′ |
| *CaAMP1* (qRT) | [AAT35532.1](https://www.ncbi.nlm.nih.gov/protein/AAT35532.1?report=genbank&log$=prottop&blast_rank=1&RID=C5PFZSJF01R) | 5′-GGTCATTGGTTAGGCGGTTTG-3′ |
|  |  | 5′-ACTCGTTGTTGCAGGGAGGC-3′ |
| *GmActin* | U60500 | 5′-GAGCTATGAATTGCCTGATGG-3′ |
|  |  | 5′-CGTTTCATGAATTCCAGTAGC-3′ |
| *GmNPR1-1* | FJ418594 | 5′- GGGGATGCCTGTATGTCTTC-3′ |
|  |  | 5′-CGCAGAAAGACCAGCAAACT-3′ |
| *GmNPR1-2* | FJ418596 | 5′-GTTGACAGTGTGTGTGCCCA-3′ |
|  |  | 5′-AACAGTGAGGATTGGGATGACA-3′ |
| *GmSGT1* | NM_001249656 | 5′-TGAGGCTGTGGCTGATGCTA-3′ |
|  |  | 5′-ACCTCCAGAGCAGCCTTTG-3′ |
| *GmRAR1* | FJ222386 | 5′-TGCTCCGAAACCTAAGAAGATA-3′ |
|  |  | R: 5′-ATCACAGCACTTCCACCCTC-3′ |
| *GmPR1* | AF136636 | 5′-TGTGTTGTGTTTGTTAGGGTTAGTCA-3′ |
|  |  | 5′-TGTTGGTGAGTCTTGAGCATACG-3′ |
| *GmPR2* | M37753 | 5′-GTCTCCTTCGGTGGTAGTG-3′ |
|  |  | 5′-ACCCTCCTCCTGCTTTCTC-3′ |
| *GmPR3* | AF202731 | 5′-GCACTTGGTCTGGATTTG-39′ |
|  |  | 5′-GGCTTGATGGCTTGTTTC-3′ |
| *GmPR5* | BU765509 | 5′-GCGCTTGCTCCGCTTTCAACT-3′ |
|  |  | 5′-CTTGGAATAGACGGTGGGCTTGC-3′ |
| *GmPR12* | BU964598 | 5′-CATGGACAAGGCACGATTTGG-3′ |
|  |  | 5′-AACCGATGGCTCTTTGACTCAC-3′ |
| *GmPAL* | X52953 | 5′-GTGCAAGGGCTGCTTATG-3′ |
|  |  | 5′-CCCAGTCCCTAATTCCTCTC-3′ |
| *GmAOS* | DQ288260 | 5′-CCTCTGTCTCCGAGAAACC-3′ |
|  |  | 5′-CCTTCAAGGGACCGATCAC-3′ |
| *GmPPO* | EF158428 | 5′-GGGTTGGTGCTGCTGATAAG-3′ |
|  |  | 5′-CGATCCGAGTTCGTGTGATG-3′ |
